# Linear mixed-effects models to describe length-weight relationships for yellow croaker (*Larimichthys Polyactis*) along the north coast of China

**DOI:** 10.1371/journal.pone.0171811

**Published:** 2017-02-22

**Authors:** Qiuyun Ma, Yan Jiao, Yiping Ren

**Affiliations:** 1 Fisheries College, Ocean University of China, Qingdao, Shandong, China; 2 Department of Fish and Wildlife Conservation, Virginia Polytechnic Institute and State University, Blacksburg, Virginia, United States of America; Xiamen University, CHINA

## Abstract

In this study, length-weight relationships and relative condition factors were analyzed for Yellow Croaker (*Larimichthys polyactis*) along the north coast of China. Data covered six regions from north to south: Yellow River Estuary, Coastal Waters of Northern Shandong, Jiaozhou Bay, Coastal Waters of Qingdao, Haizhou Bay, and South Yellow Sea. In total 3,275 individuals were collected during six years (2008, 2011–2015). One generalized linear model, two simply linear models and nine linear mixed effect models that applied the effects from regions and/or years to coefficient *a* and/or the exponent *b* were studied and compared. Among these twelve models, the linear mixed effect model with random effects from both regions and years fit the data best, with lowest Akaike information criterion value and mean absolute error. In this model, the estimated *a* was 0.0192, with 95% confidence interval 0.0178~0.0308, and the estimated exponent *b* was 2.917 with 95% confidence interval 2.731~2.945. Estimates for *a* and *b* with the random effects in intercept and coefficient from Region and Year, ranged from 0.013 to 0.023 and from 2.835 to 3.017, respectively. Both regions and years had effects on parameters *a* and *b*, while the effects from years were shown to be much larger than those from regions. Except for Coastal Waters of Northern Shandong, *a* decreased from north to south. Condition factors relative to reference years of 1960, 1986, 2005, 2007, 2008~2009 and 2010 revealed that the body shape of Yellow Croaker became thinner in recent years. Furthermore relative condition factors varied among months, years, regions and length. The values of *a* and relative condition factors decreased, when the environmental pollution became worse, therefore, length-weight relationships could be an indicator for the environment quality. Results from this study provided basic description of current condition of Yellow Croaker along the north coast of China.

## Introduction

Length-weight relationships (LWR) are used for estimating the weight corresponding to a given length [1]. As most observations in fisheries surveys are typically obtained as the number of specimens and length of each sampled specimen, LWR are widely used to transform the survey data into estimates of the biomass which is important for modelling aquatic ecosystems. Therefore, estimation of the LWR is common practice and essential in fisheries science [2].

There are two parameters in the LWR model (*W* = *a*×*L*^*b*^), the coefficient *a* and the exponent *b*. Parameter *a* is the condition factor, describing body shape which can be classified as four groups: eel-like, elongated, fusiform and short and deep [2]. Parameter *b* is the allometric growth parameter, which indicates isometric growth in body proportions if *b*≥3 where fish have more girth as it grows longer; the species tends to be more streamlined if the exponent *b*<3 [2].

Numerous studies found that factors, such as geographic, seasonal, inter-annual, and environmental conditions, can affect the estimates of *a* and *b* in the LWR model [2–5]. Instead of constructing several models reflecting various situations (different regions and years), it is more reasonable to make use of generalized linear mixed model to cover all the spatial and temporal effects in a single model. Linear mixed-effects modelling, a mature model in the statistics community, has been used in multiple fields. Cnaan et al. (1997) provided two detailed case studies to sufficiently introduce the use of the general linear mixed effects model for the regression analysis of correlated data [6]. Xu et al. (2015, 2014) developed nonlinear mixed-effects model to study the individual-tree diameter growth and linear mixed effects model for individual-tree crown width of China-Fir trees in Southeast China [7, 8]. Baayen et al. (2008) provided an introduction of mixed-effects models and illustrated its advantages, which would allow the researcher to simultaneously consider all factors that potentially contribute to the understanding of the structure of the data [9].

The linear mixed-effects model provides a powerful and versatile approach to analyze a wide variety of data structures, in which the linear predictor contains random effects in addition to the usual fixed effects [7]. The random effects vary with respect to one or more grouping variables, e.g. regions and years, adding its contribution of variation to the residual error, which can account for the additional resources of random variation [7, 8, 10].

Yellow Croaker (*Larimichthys polyactis*) is a warm-temperate demersal fish species widely distributed throughout the northwest Pacific Ocean. As a commercially important species, Yellow Croaker experienced heavy fishing pressure in China. From catch data derived from *China Fishery Statistical Yearbook*, we found the variations of Yellow Croaker stock (S1 Fig). Yellow Croaker were abundant in 1950s and the beginning of 1960s; but the stock collapsed in the 1970s [11]. After a series of restoration efforts (i.e. seasonal fishery closure and protection of the spawning ground), the Yellow Croaker stock has been recovering since 1990 and the catch has increased continually in the subsequent two decades [12–14]. However, the characteristics of the stock have changed; as individuals are smaller, younger and reaching maturity earlier [13,15,16].

Quite many scientists have done much work about the growth of Yellow Croaker around the world. Li *et al*. (2013) applied simple linear regression to analyze LWRs of Yellow Croaker in Bohai Sea-Northern Yellow Sea from 1960 to 2004 and in the Southern Yellow Sea from 1960 to 2010 for male and female respectively [3]. Zhang *et al*. (2010) investigated the biological characteristics of Yellow Croaker in the central and southern Yellow Sea, including the LWRs in 1960, 1985, 1998 and 2008 [17]. Lin *et al*. (2004) studied the population biology of Yellow croaker in the East China Sea, which evaluated the LWR in 1963, 1983 and 2001 [18]. All these studies of LWR used simple linear regression to develop region specific or year specific models, rather than linear mixed-effects models, to detect the spatial and temporal variations of Yellow Croaker.

In this study, our objective was to analyze the biological characteristics of Yellow Croaker in recent years. Specifically, we estimated the LWR of Yellow Croaker used linear mixed-effects model, condition factors relative to reference years, and explore the possible relationship between parameters in LWR model and environmental factors, based on the observations along north coast of China among 2008 and 2011 to 2015.

## Materials and methods

### Data collection

**Fig 1:**
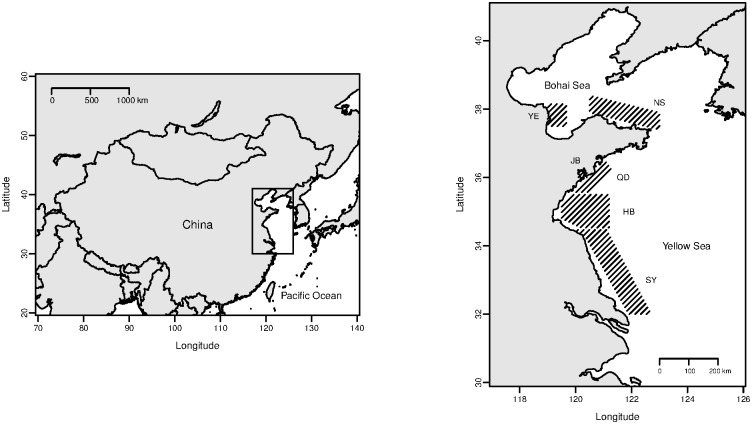
Survey map of Yellow Croaker along the north coast of China. The shades in the right plot showed the surveying area. Abbreviations of regions were detailed in Table 1.

Specimens were collected along the Shandong and Jiangsu province through 2008, and 2011–2015. These regions along northern Chinese coast include Yellow River Estuary (YE), Coastal Waters of Northern Shandong (NS), Jiaozhou Bay (JB), Coastal Waters of Qingdao (QD), Haizhou Bay (HB), and South Yellow Sea (SY) (Table 1, S1 Table, Fig 1), which are important spawning and feeding grounds of Yellow Croaker [19]. The maps of surveying area were plotted using package maps and mapdata of the R (version: R i386 3.3.1) [20, 21].

**Table 1:** Sample size and location of Yellow Croaker among regions and years.

Regions		2008	2011	2012	2013	2014	2015	Total
Yellow River Estuary	YE				40			40
Coastal Waters of Northern Shandong	NS					35		35
Jiaozhou Bay	JB	426	26	63				515
Coastal Waters of Qingdao	QD			419		533		952
Haizhou Bay	HB		921		81	100	579	1681
South Yellow Sea	SY					148	11	308
**Total**		426	947	482	121	816	590	3382

The second column shows the abbreviations of regions.

In Coastal waters of Northern Shandong, specimens were randomly collected from fishermen immediately after landing. For all other five regions, stratified random bottom trawling surveys were implemented to collect samples. In total, 3,275 individuals were collected, with sample size variations among years and regions (see Table 1 and S1 Table). For each individual, standard length was measured to the nearest mm and total weight was measured to the nearest gram.

The surveys, which held in the *Seasonal Fishery Closure* (June, July and August) were approved by the Department of Marine and Fishery of Shandong and Jiangsu Province. No specific permissions were required for all the other surveys, because these surveys are traditional surveys, which did not cover any marine protected area or private area and this study did not involve endangered or protected species.

### LWR modelling

We fitted an exponential function to the weight at length data that took the form [1]:

$$W=aL^{b}$$

where *W* is the wet weight of an individual fish (g), *L* is the standard length (cm), *a* is the condition factor, and *b* is the allometric growth parameter. Because the variance of W increases when L increases, above equation was log-transformed and the equation becomes:

ln(W)=ln(a)+b×ln(L)

We fit a generalized linear model (GLM), simple linear models for individuals in different regions and years (SLMR and SLMY for regions and years respectively), as well as nine linear mixed effect models (LMEM) that treated region and/or year as random effects to coefficient *a* and/or the exponent *b* to explain the relationship between length and weight (Table 2) [7]. Analysis of Variance performed by *F*-test between GLM and LMEM was computered to tests whether the temporal and spatial variations were significant. All these modelling processes were conducted, using package lme4 the R (version: R i386 3.3.1) [22]. Bootstrap the data with replacement for 1000 times was used to estimate the distributions and statistics of parameter estimates in these models.

**Table 2:** Models for length-weight relationships of Yellow Croaker.

	Models	Log-Transformed	AIC	ΔAIC	MAE
GLM	W = a*L^*b*	ln(W) = ln(*a*)+b*ln(L)	-3777	581	0.106
SLMR	W = *a*_i_*L^*b*_*i*_	ln(W) = ln(*a*_*i*_)+*b*_i_*ln(L)	-4002	356	0.097
SLMY	W = *a*_j_*L^*b*_*j*_	ln(W) = ln(*a*_*j*_)+*b*_j_*ln(L)	-4254	104	0.102
R.I	W = (a*exp(ReR.I))*L^*b*	ln(W) = (ln(*a*)+ReR.I)+b*ln(L)	-4005	353	0.102
Y.I	W = (a*exp(ReY.I))*L^*b*	ln(W) = (ln(*a*)+ReY.I)+b*ln(L)	-4220	138	0.098
R&Y.I	W = (a* exp(ReR.I)* exp(ReY.I))*L^*b*	ln(W) = (ln(*a*)+ReR.I+ReY.I)+b*ln(L)	-4341	17	0.098
R.S	W = a*L^(*b*+ ReR.S)	ln(W) = ln(*a*)+(*b*+ReR.S)*ln(L)	-3999	359	0.102
Y.S	W = a*L^(*b*+ ReY.S)	ln(W) = ln(*a*)+(*b*+ReY.S)*ln(L)	-4212	146	0.098
R&Y.S	W = a*L^(*b*+ReR.S+ReY.S)	ln(W) = ln(*a*)+(*b*+ReR.S+ReY.S)*ln(L)	-4334	24	0.098
R.I&S	W = (a* exp(ReR.I))*L^(*b*+ReR.S)	ln(W) = (ln(*a*)+ReR.I)+(*b*+ReR.S)*ln(L)	-4009	349	0.102
Y.I&S	W = (a* exp(ReY.I))*L^(*b*+ReY.S)	ln(W) = (ln(*a*)+ReY.I)+(*b*+ReY.S)*ln(L)	-4267	91	0.097
R&Y.I&S	W = (a* exp(ReR.I)* exp(ReY.I))*L^(*b*+ReR.S+ReY.S)	ln(W) = (ln(*a*)+ReR.I+ReY.I)+(*b*+ReR.S+ReY.S)*ln(L)	-4358		0.095

The first column shows the abbreviations of models detailed in the second and third columns.

L: standard length in centimeters; W: whole weight.

R.I: random effects on intercept (ln(*a*)) of Regions (HB, JB, NS, QD, SY, and YE);

R.S: random effects on slope (*b*) of Regions;

Y.I: random effects on intercept (ln(*a*)) of Years (2008, 2011, 2012, 2013, 2014, and 2015);

Y.S: random effects on slope (*b*) of Years.

*i* is the *i*th region; *j* is the *j*th year.

ΔAIC = AIC of models—AIC of best fitted model (R&Y.I&S).

We explored the influence of marine environmental status on variability of the LWR. Environmental data were drawn from *Bulletin of Marine Environmental Status of China*, which were reported by State Oceanic Administration People’s Republic of China [23]. According to *Sea Water Quality Standard* (National Standard GB 3097–1997) [24], five levels (the first, second, third, fourth and worse than fourth levels) were used to evaluate the water quality, in which higher level indicated poorer quality. The polluted water area percentage (PWAP), which included the third, fourth and worse than fourth level water, was used to exemplify water quality. We explored the influence of PWAP on the LWR along north coast of China during 2008, 2011 to 2015.

### Model comparison

Both the Akaike information criterion (AIC) and the mean absolute error (MAE) were calculated to compare the performance of the twelve candidate models. AIC was widely used to compare the quality of models [25,26]. Lower AIC value indicates a better model. MAE is a quantity used as a measurement on how close forecasts or predictions are to the eventual outcomes [27].

MAE was the average of the absolute errors:

$$MAE=\frac{1}{n}\overset{n}{\underset{i=1}{\sum }}\left(\left|f_{i}-y_{i}\right|\right)$$

where *f*_*i*_ was the estimation and *y*_*i*_ the observed value.

### Relative condition factor

Relative condition factor (*K*) was calculated for each individual [28]:

$$K=\frac{W}{aL^{b}}$$

where *W* and *L* were observed weight and standard length from our surveys, baseline model LWR with parameters *a* and *b* were drawn from historical references. Relative condition factor was recommended to measure the deviation of an individual from the baseline weight for length in the respective sample and to investigate changes in the stock over time [2,28]. Here the LWR models of Yellow Croaker in 1960, 1986, 2005, 2007, 2008.04~2009.03 (12 months) and 2010 were introduced to be the baselines (S2 Table) [3,11]. In 1960, Yellow Croaker were abundant and the stock was not deeply disturbed by fishing [13]. However, Yellow Croaker experienced severe fishing pressure and reached its lowest biomass in 1986 [14]; after recovery, the catch of the stock was increasing in recent years [12,14]. The current condition factors relative to reference years of 1960, 1986, 2005, 2007, 2008~2009 and 2010 were represented by *K*_*cur/1960*_, *K*_*cur/1986*_, *K*_*cur/2005*_, *K*_*cur/2007*_, *K*_*cur/2008 9*_ and *K*_*cur/2010*_.

## Results

### Length and weight of Yellow Croaker

**Fig 2:**
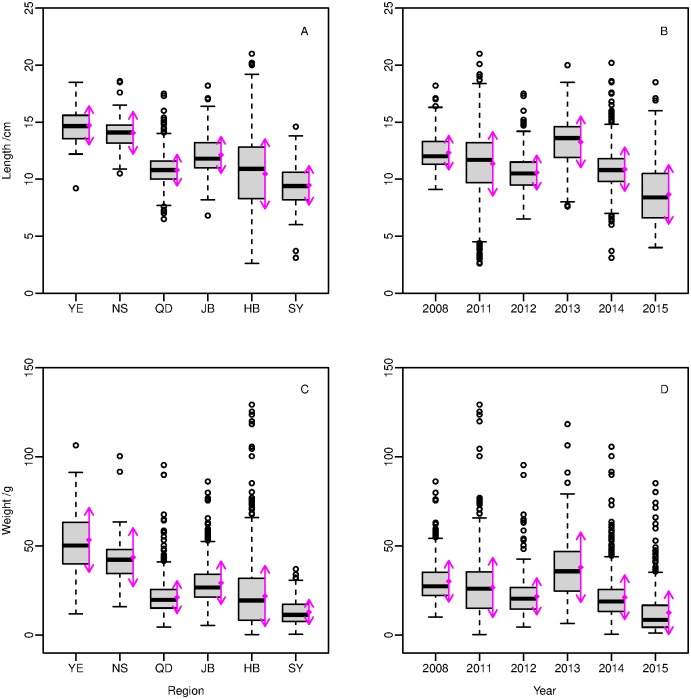
Length and weight of Yellow Croaker among regions and years. The boxes show the median (solid line) and the interquartile range. The plus symbols are indications of outliers in the data. Arrows in magenta show the mean and standard deviation. Abbreviations of regions were detailed in Table 1.

The length frequency of collected samples of Yellow Croaker is shown in S2 Fig. The maximum, minimum and mean (95% CI) lengths of the Yellow Croaker sample were 21.0 cm, 2.6 cm and 10.9 cm (5.1, 15.5 cm), respectively. The most commonly encountered length group was 11.5–12.4 cm, followed by length group 10.5–11.4 cm. The sample was dominated by individuals ranging from 8 cm to 14 cm in length, with variations among regions and years (Fig 2).

Lengths of most individuals in JB, QD, NS, and YE were longer than 10 cm, ranging from 8–14 cm in HB and 6–12 cm in SY. Both the length and the weight had a positive relationship with latitude, where larger individuals were more common in northern than southern regions. Most lengths in 2008, 2011 and 2013 were longer than 10 cm and within 8–14 cm in 2012 and 2014, but 5–12 cm in 2015. Individuals in 2013 were larger than fish collected in other years, while a third of these specimen caught in Yellow River Estuary. All specimens were collected in the south area (Haizhou Bay and Southern Yellow Sea) in 2015 and were noticeably smaller in length and weight (Fig 2), might because of individual growth or the different age groups among specimens in different regions.

### Recommended LWR

The LMEM (R&Y.I&S) with Regions and Years have random effects on both intercept and slope was recommended to be used as the best model after comparing with the other models based on AIC and MAE values (-4357 and 0.095, respectively) (Table 2). According to this selected model, *a* and *b* were estimated as 0.0192 (95% CI = 0.0178, 0.0308) and 2.917 (95% CI = 2.731, 2.945), respectively (Table 3). The estimates of *a* with the random effects from Region and Year ranged from 0.013–0.023, while the estimates of *b* with the random effects from Region and Year ranged from 2.835–3.017.

**Table 3:** Estimated values of parameter *a* and *b* in GLM and LEMM models.

Models	*a*				*b*			
	Best	Bootstrap			Best	Bootstrap		
		Mean	95% CI	Std.		Mean	95% CI	Std.
GLM	0.0170	0.0170	(0.0163, 0.0177)	0.00035	2.961	2.961	(2.944, 2.979)	0.009
R.I	0.0180	0.0180	(0.0171, 0.0189)	0.00047	2.934	2.934	(2.913, 2.956)	0.011
Y.I	0.0180	0.0181	(0.0172, 0.019)	0.00046	2.936	2.936	(2.915, 2.956)	0.011
R&Y.I	0.0189	0.0189	(0.0180, 0.0199)	0.00049	2.921	2.921	(2.900, 2.941)	0.010
R.S	0.0178	0.0178	(0.0170, 0.0187)	0.00043	2.938	2.938	(2.918, 2.957)	0.010
Y.S	0.0179	0.0179	(0.0170, 0.0188)	0.00045	2.940	2.940	(2.919, 2.960)	0.011
R&Y.S	0.0184	0.0184	(0.0175, 0.0193)	0.00047	2.930	2.930	(2.909, 2.953)	0.011
R.I&S	0.0195	0.0205	(0.0179, 0.0269)	0.00234	2.906	2.887	(2.783, 2.938)	0.039
Y.I&S	0.0176	0.0176	(0.0165, 0.0188)	0.00060	2.947	2.947	(2.920, 2.973)	0.014
R&Y.I&S	0.0192	0.0218	(0.0178, 0.0308)	0.00362	2.917	2.871	(2.731, 2.945)	0.058

Estimates in the best fitted model; mean, 95% confidence interval and standard deviation got from models after bootstrap the data.

### Spatial and temporal variations of LWR

**Fig 3:**
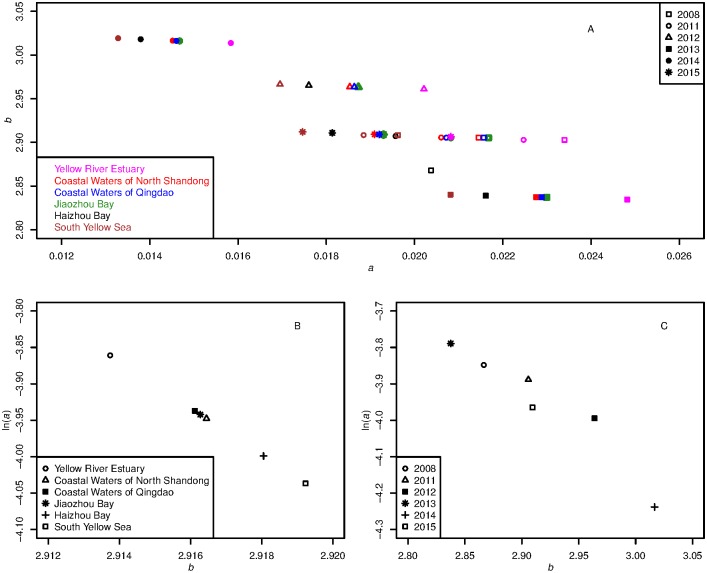
Variations of *a* and *b* among regions and years from the LMEM (R&Y.I&S). A: estimates of parameter *a* and *b* with random effects of both regions (presented in various colors) and years (presented in various markers). B: spatial variations of ln(*a*) and *b*. C: temporal variations of ln(*a*) and *b*.

The selected model with random effects from both regions and years applied to both *a* and *b*, indicated that there were substantial spatial and temporal variations of LWR for Yellow Croaker (Table 4, Fig 3, S3 and S4 Figs). The results of analysis of vriance between the selected LMEM and the global model GLM suggested that the spatial and temporal variations of LWR of Yellow Croaker are significant (F = 111434, Df = 1, *P*<0.001). The *a* value in the length-weight model, decreased with latitude (32.75°-38.15° N), while the parameter *b* value increased with latitude. The estimates for Coastal Waters of Qingdao and Jiaozhou Bay were similar, while their latitudes covered 35.33°-36.49° N and 35.99°-36.13° N, respectively. Variations among regions were 0.0034 and 0.0055 for *a* and *b*, respectively; but were larger among years (0.0081 and 0.1791 for *a* and *b*, respectively).

**Table 4:** Estimates values for parameters *a* and *b* in the LMEM (R&Y.I&S).

	Effects	ln(*a*)	*a*	*b*
Fixed effects		-3.954	0.019	2.917
Random Effects	Yellow River Estuary	0.093	1.098	-0.0029
	Coastal Waters of Northern Shandong	0.006	1.006	-0.0002
	Coastal Waters of Qingdao	0.017	1.017	-0.0005
	Jiaozhou Bay	0.012	1.012	-0.0004
	Haizhou Bay	-0.045	0.956	0.0014
	South Yellow Sea	-0.083	0.920	0.0026
	2008	0.106	1.111	-0.0500
	2011	0.065	1.068	-0.0109
	2012	-0.041	0.960	0.0472
	2013	0.165	1.179	-0.0791
	2014	-0.284	0.752	0.1000
	2015	-0.011	0.989	-0.0072

Fixed values of ln(*a*), *a* and *b*; random effects from six regions and six years respectively.

Instead of a gradual trend of spatial variation, temporal variation was out of order, with complex influence. The estimate of *a* reached its maximum value (0.022) in 2013, declined by 0.001 through 2008, 2011, 2015 and 2012, and had its minimum value (0.014) in 2014. The largest estimate of parameter *b* appeared in 2012, followed by the estimate (2.96) in 2012, while the minimum estimates was 2.84 in 2013.

**Fig 4:**
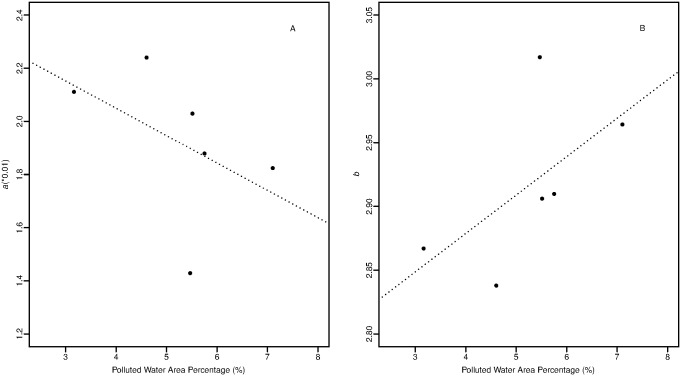
Water quality versus parameters *a* (A) and *b* (B) in LWR of Yellow Croaker.

In Yellow Sea, the PWAP occupied 3.2~7.1 percentage of the whole area during the survey time, while the PWAP covered 17.7~37.2% of the Bohai. In addition, the water quality of Chinese coast varied widely among years. When the water quality became poorer, condition factor *a* decreased, with the reverse condition for parameter *b* (Fig 4). Negative correlation (-0.47) and positive correlation (0.61) were found for PWAP with condition factor *a* and for PWAP parameter *b* respectively, while t-test indicated both the correlation coefficients were not significant (df = 4, *P*>0.05).

### Relative condition factors

**Fig 5:**
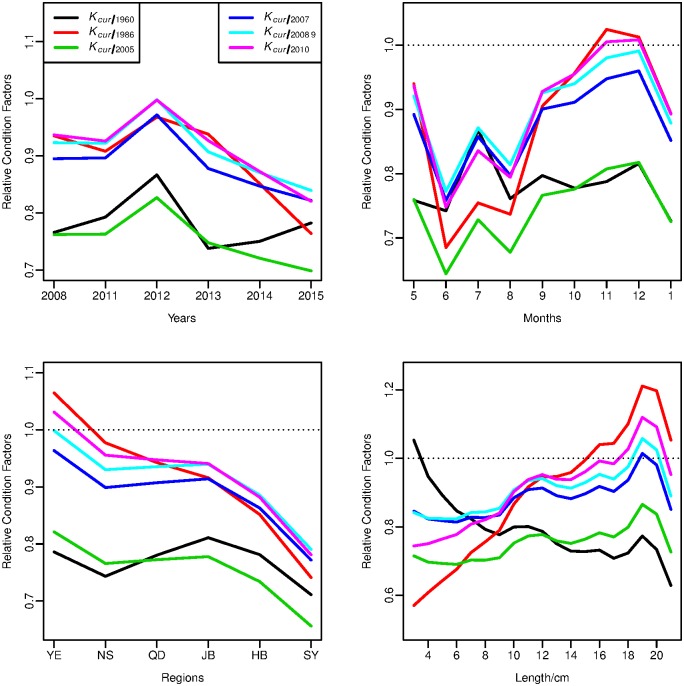
Temporal (year, month), spatial and length variations of condition factors relative to reference years of 1960, 1986, 2005, 2007, 2008~2009 and 2010. Abbreviations of regions were detailed in Table 1. The dot lines show the relative condition factor equal to 1.0.

Summary of condition factor relative to reference year of 1960, 1986, 2005, 2007, 2008~2009 and 2010 were shown in S2 Table. The mean of *K*_*cur/2005*_ was the smallest value (0.750), followed by *K*_*cur/1960*_ (0.786) and the mean estimates of *K*_*cur/2007*_ and *K*_*cur/1986*_ (0.881 and 0.882, respectively), and those of K_*cur/2008 9*_ and K_*cur/2010*_ were the highest (0.906 for both). The distributions (S5 Fig) revealed that there was more variability in *K*_*cur/1986*_, compared to others. Furthermore, variations of relative condition factors among years, months, regions and length shown in Fig 5.

**Fig 6:**
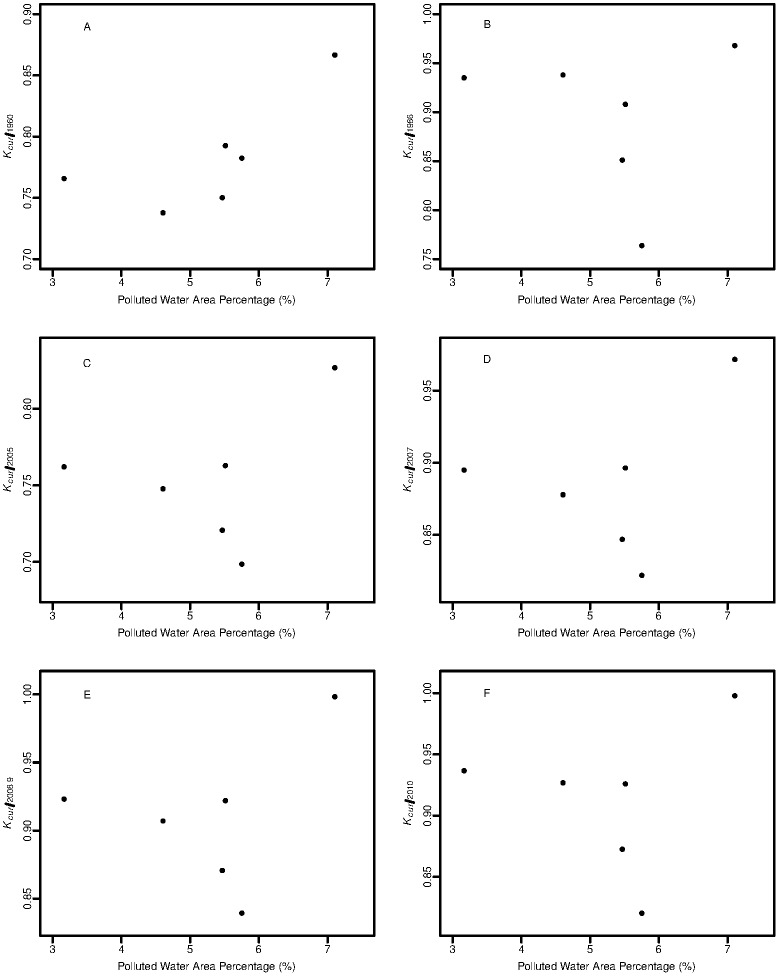
Water quality to condition factors relative to reference years of 1960 (A), 1986 (B), 2005(C), 2007 (D), 2008~2009(E) and 2010(F).

The estimates of condition factors relative to each reference year were similar in 2008 and 2010, and reached their maximum in 2012. With the exception of *K*_*cur/1960*_, relative condition factors began to decline in 2012. However, values of *K*_*cur/1960*_ remained stable over time, again with the exception of 2012. Relative condition factors were much lower in summer than other seasons, and reached maximum during autumn and early winter, with diverse variations degree to different reference years. As for spatial variations, values of relative condition factors decreased with decreasing latitude, while there was little variation (0.71~0.81) for *K*_*cur/1960*_ among different regions. Generally, relative condition factors increased as Yellow Croaker grew, except for *K*_*cur/1960*_, while there were different variation ranges among different sizes of individuals for condition factors relative to different reference year(Fig 5). When the body length of Yellow Croaker was less than 10 cm, values of condition factores relative to reference years 2005, 2007, 2008~2009 and 2010 kept much constant. However, values of *K*_*cur/1960*_, decreased as individuals grew larger, and remained stable at lengths 14–18 cm. When the water quality declined, values of *K* also decreased, while there was little variation in *K*_*cur/1960*_ (0.75~0.79), all with an exception of 2012 (Fig 6). Without data in 2012, correlation analysis indicated negative correlations for PWAP with condtion factors relative to reference years except 1960 (from -0.68 to -0.61), while no significance were found in t-test (df = 3, *P*>0.05).

## Discussion

### LWR of Yellow Croaker and its spatial-temporal variations

According to the results of LMEM (R&Y.I&S), the estimates of *a* (0.013 ~ 0.023), fell in the common range (0.001~0.05) of most fish species [2]. Study of Froese [29] shows that values of parameter *a* of fish species, with fusiform body shape, was 0.0112 (geometric mean) with 95% range of 0.00514 to 0.0245. The results are consistent with the body shape of this species, which is described as fusiform in Fishbase [30].

The point estimate of exponent *b*, was a little lower than values 3.03 (2.91–3.15) in Fishbase [29]. Values of parameter *b* for fishes were suggested by Carlander [31] and Froese [2] to normally fall within the range of 2.5 ~ 3.5 and 2.7 ~ 3.4, respectively, which cover the range of parameter *b* of Yellow Croaker in this study. The fixed value of allometric growth parameter *b*, indicated a very slight decrease in plumpness or elongation in form with increase in length. However, when random effects were used, no significant difference with isomeric growth was found for Yellow Croaker [32].

Random region effects for *a* demonstrated that values of *a* reduced gradually as the latitude decreased, from north to south, except in Coastal Waters of Northern Shandong. Samples in the Coastal Waters of Northern Shandong were collected from fishermen, and the origin of the samples were not clear, which could be the reason that parameters with the random effect of NS did not follow this trend. Yellow Croaker distributed on China’s coast were considered to be divided into various populations, while individuals in northern Yellow Sea and Bohai Sea (NYBS) were believed to be different from those in southern Yellow Sea (SYS) [19,33,34]. The results of this study suggested that Yellow Croaker sampled from Yellow River Estuary and Coastal Waters of Northern Shandong should be included in NYBS, while those in other regions belonged to SYS. Different subpopulation maybe the main reason that led to their significant spatial variation in length-weight relationships.

Previous research has examined LWR variation for Yellow Croaker; including spatial, temporal, sexual, and length variations, which covered Bohai Sea, Yellow Sea and East China Sea from 1960 to 2010 [3,11,14,17,18, 35–41]. In these LWRs (51 models), values of parameter *a* ranged from 0.0061 to 0.1027, with mean of 0.028 ± 0.019; values of exponent *b* ranged from 2.32 to 3.35, with mean of 2.875 ± 0.217. This, accompanied with relative condition factors, could support the hypothesis, that individuals were becoming thinner and young-age in recent years.

Several influencing factors of LWR were evaluated in previous studies [3,42,43]. Pollutions such as inorganic nitrogen, active phosphate and oil pollutant, frequent pollutants in the north coast of China, are believed to make food availability and oxygen level deteriorate, which would negatively influence the growth of Yellow Croaker [44–46]. In this study, the data revealed that values of condition factor *a* decreased, when the environmental pollution increased. Therefore, LWR could be a rough indicator for the environment quality. Limited sample years maybe the reason that resulted in the non-significant correlation from statistics test, so further long term monitoring is suggested in the future.

### Condition changes compared with historical records

Relative condition factor provided an effective approach to compare the observed weight of an individual with the baseline weight of that length [28,47]. Compared to individuals in previous decades [3,11], Yellow Croaker condition declined in recent years. *K*_*cur/1960*_ remained comparatively stable, without obvious variation. *K*_*cur/2005*_, *K*_*cur/2007*_, *K*_*cur/2008 9*_ and *K*_*2010*_ had similar variation patterns, since the LWR of Yellow Croaker in these years were similar, while values of *K*_*cur/2005*_ were much lower. However, *K*_*cur/1986*_ have similar variation pattern but higher variation, since the stock of Yellow Croaker collapsed during the 1980s and had been in an unstable condition in 1986 [16,48].

Condition factors relative to all reference years, reached the highest value in 2012, as specimens in 2012 were collected in May and September (S1 Table), which was just before spawning and after feeding. A weak El Nino in 2012 might be another possible reason that led to elevated relative condition factors.

Monthly changes in condition factors are related to life history events of this fish, e.g., sexual maturation, spawning and feeding strategies [14,49,50]. Adult yellow croaker in these areas reach their full gonads maturity in spring, reproduce in June and then primarily feed in summer and autumn [49,51]. The condition factor (*K*) behaved in accordance to this series of events. It reached a high value in May, dropped precipitately in June, gradually recovered in August and reached the highest value in December.

Spatial variation of *K* discovered that Yellow Croaker was skinny in the south, with worse condition than in the north. It was widely reported the Yellow Croaker stock shifted in size and age structure to feature more small and young fish that mature earlier, in response to severe fishing pressure over the past half century [15,16,40,48]. This circumstance was also reflected in the length variation of condition factor relative to reference year of 1960.

Since there was only one sample of L = 21cm, the condition factor relative to all reference years became an exception in L = 21cm for length variation of Yellow Croaker. Moreover, just as values of parameter *a*, water pollution led to *K* worse. The exception value *K* in 2012 relative to all reference years, probably resulted from the more significant effects from monthly variation and nearly null El Nino in 2012 (Fig 5).

### Applications of mixed effects model in studying spatial-temporal variations of LWR

According to AIC and MAE, linear mixed effect model with effects from both regions and years on both parameters *a* and *b* was supported as the best one. The recommended model matches previous studies that the exact relationship between length and weight differs among regions and years, according to the habitat condition [4,5,52,53]. However, many studies on LWR for fishes used region specific or year specific models fitting to region or year specific dataset to detect the spatial and temporal variations of individuals [4,54,55], as seen from previous 51 LWR models for Yellow Croaker [3,11,14,17,18,35–41]. The mixed effects model took account of regions and years as random effects in a single model, which was more convenient and reasonable to estimate the spatial and temporal variations. It also allow regions and years with limited samples to borrow strength from other regions and years [10].

Furthermore, mixed effects model provided an effective approach to estimate the effects of variables, observations of which could be unavailable [7,8]. In this study, our survey data were quite poor: there were 23 blanks in the 6×6 (Regions-Years) data matrix (Table 1), while mixed effects model effectively evaluated the effects of regions and years. In the future, more variations of LWR, such as sex, season, and growth stage should be considered in the mixed-effects model for Yellow Croaker.

## Conclusions

According to this study, the condition of Yellow Croaker declined in recent years and was skinny in the south, with worse condition than in the north. The values of condition factor *a* in LWR decreased, when the environmental pollution increased. Therefore, LWR could be a rough indicator for the environment quality. The mixed effects model provided a more convenient and reasonable approach to estimate the spatial and temporal variations of LWR for Yellow Croaker.

## Supporting information

S1 Fig Yield of Yellow Croaker in China. Data were derived from *China Fishery Statistical Yearbook* from 1956 to 2012.
(TIF)

S2 Fig Length frequency of Yellow Croaker. (TIF)

S3 Fig LWR differences of Yellow Croaker among regions and years from the LMEM (R&Y.I&S). a, b, and c were plots among regions; d, e, and f were plots among years. a and d plots cover the whole range of length; b and e plots cover the median part (Length = 12–16 cm); c and f plots cover the large size (Length = 16–22 cm).
(TIF)

S4 Fig Parameters *a* and *b* distributions among regions and years. A and B plots were parameters variation among regions, with C and D plots among years. B and D plots zoom the main parts with high frequency.
(TIF)

S5 Fig Distribution of condition factors relative to reference years of 1960 (A), 1986 (B), 2005(C), 2007 (D), 2008~2009(E) and 2010(F). (TIF)

S1 Table Monthly sample size of Yellow Croaker among regions and years. The detailed information of regions presents in Table 1; no individuals were caught during February, March and April.
(DOCX)

S2 Table Condition factors of Yellow Croaker relative to reference year of 1960, 1986 and 2007, and the references LWR models. See the text for the definition of Kcur/1960, Kcur/1960 and Kcur/2007.
(DOCX)
