# Supplementary figures and images for: Linear mixed-effects models to describe length-weight relationships for yellow croaker (Larimichthys Polyactis) along the north coast of China
Source: PLoS One. 2017 Feb 22;12(2):e0171811. doi: 10.1371/journal.pone.0171811 (PMC5321278; doi:10.1371/journal.pone.0171811)

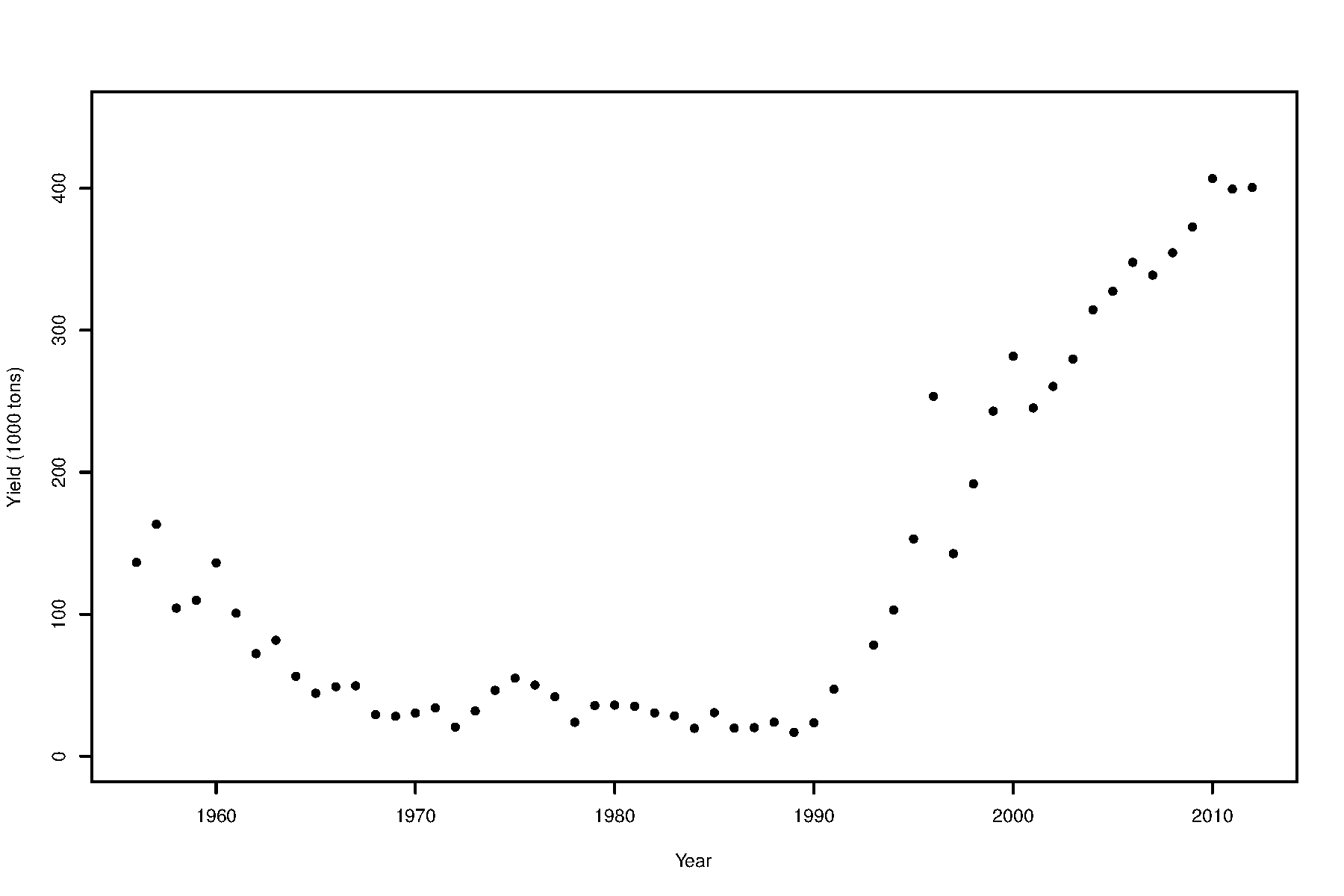

Supplement: S1 Fig — Data were derived from China Fishery Statistical Yearbook from 1956 to 2012. (TIF) [file pone.0171811.s001.tif]

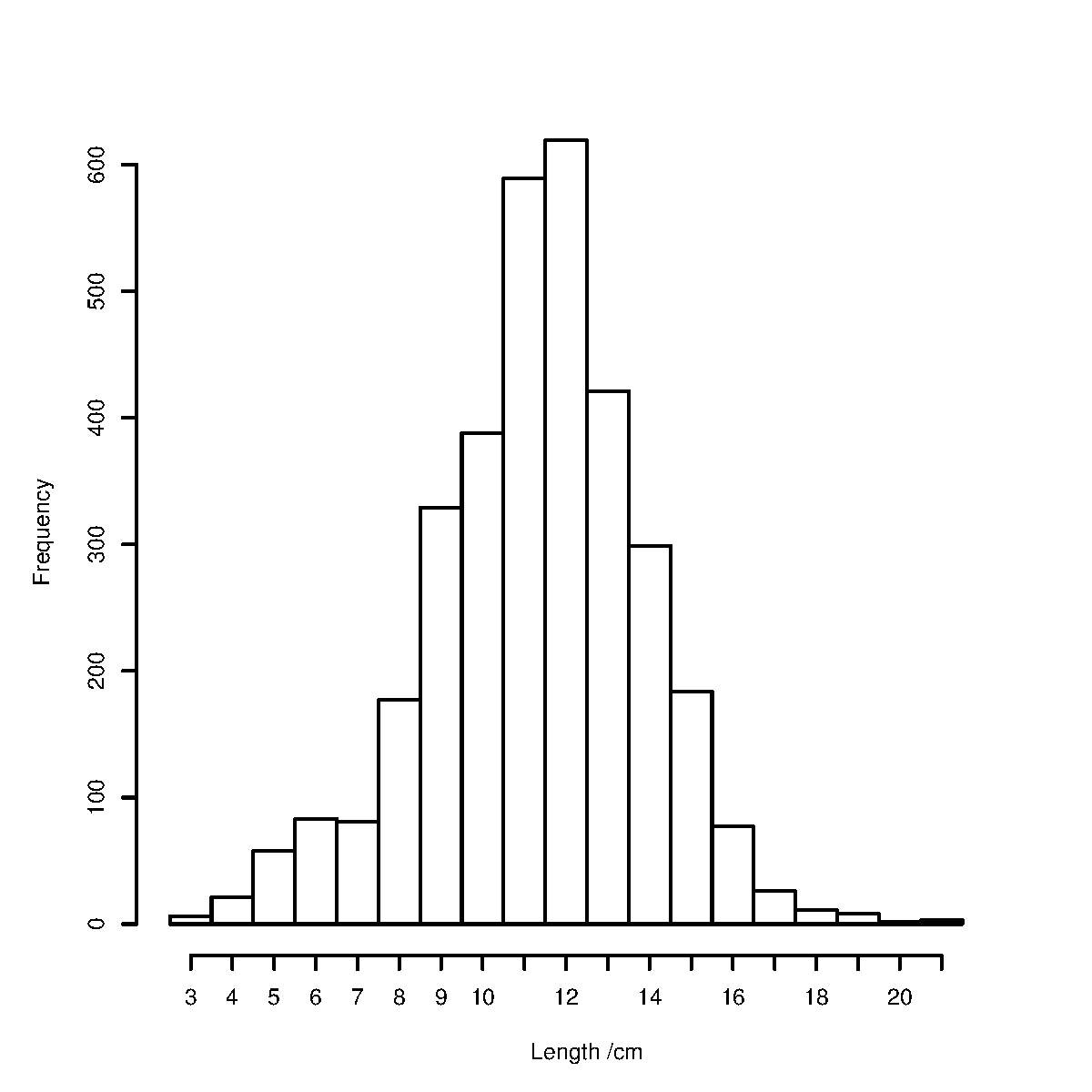

Supplement: S2 Fig — (TIF) [file pone.0171811.s002.tif]

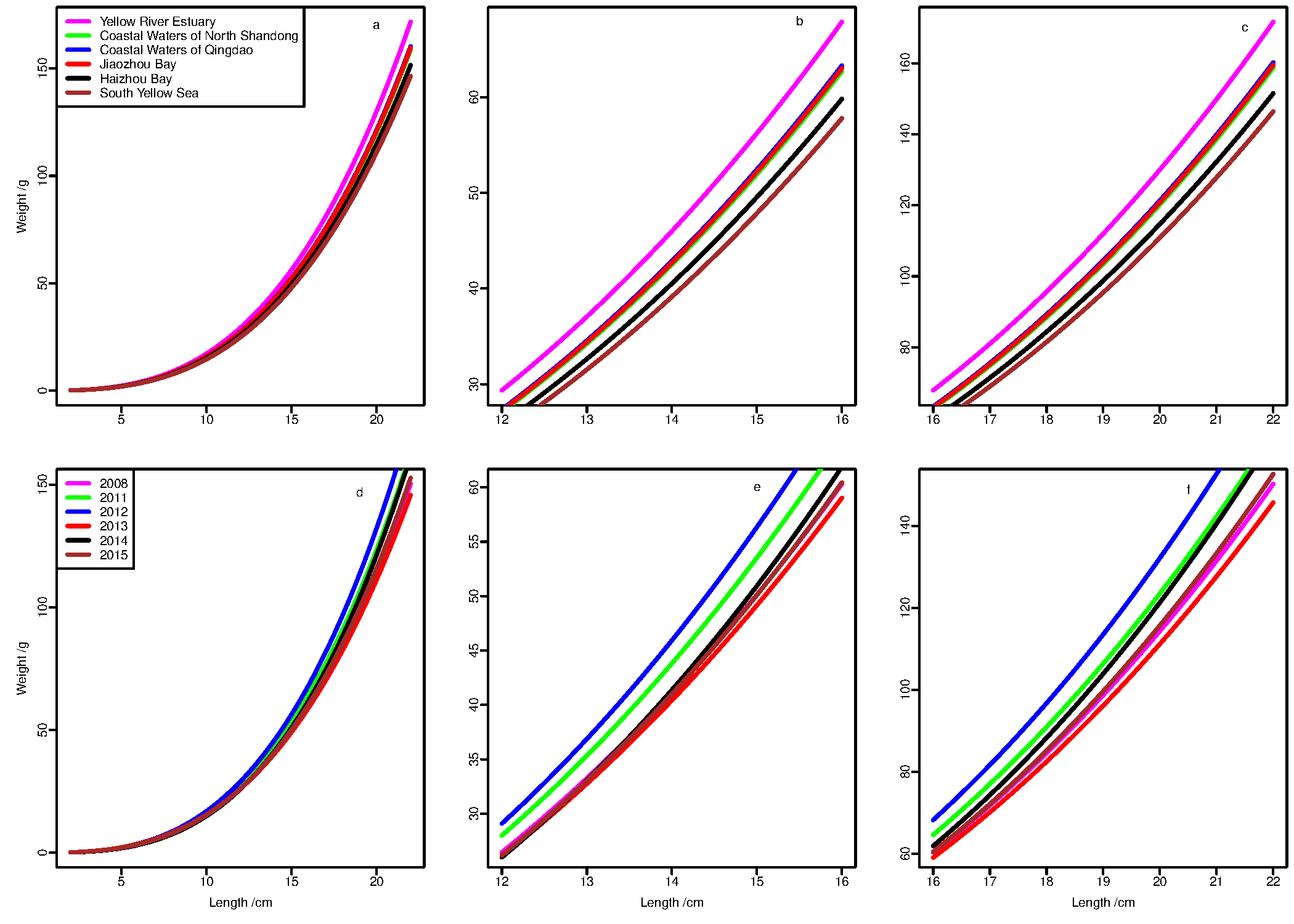

Supplement: S3 Fig — a, b, and c were plots among regions; d, e, and f were plots among years. a and d plots cover the whole range of length; b and e plots cover the median part (Length = 12–16 cm); c and f plots cover the large size (Length = 16–22 cm). (TIF) [file pone.0171811.s003.tif]

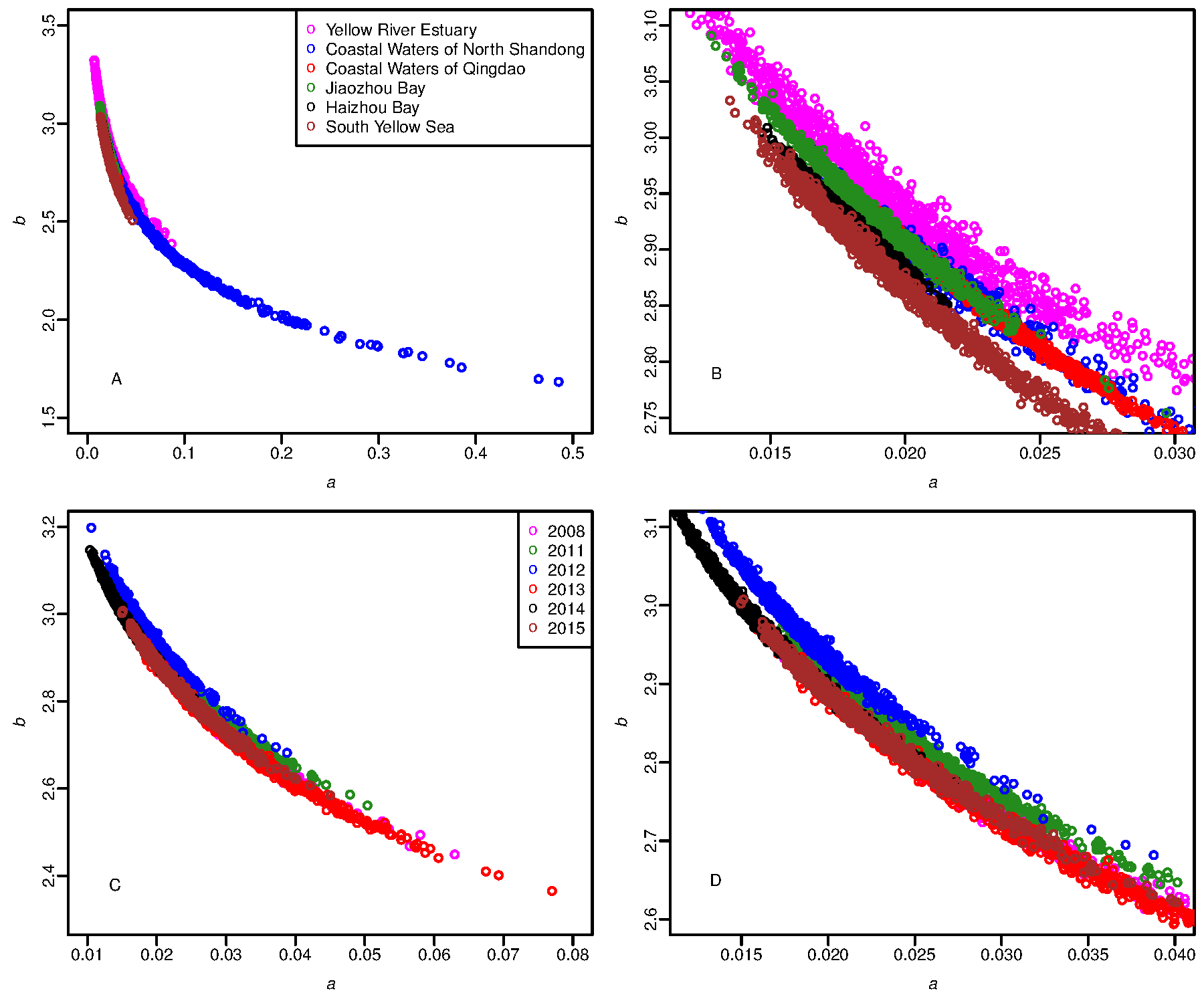

Supplement: S4 Fig — A and B plots were parameters variation among regions, with C and D plots among years. B and D plots zoom the main parts with high frequency. (TIF) [file pone.0171811.s004.tif]

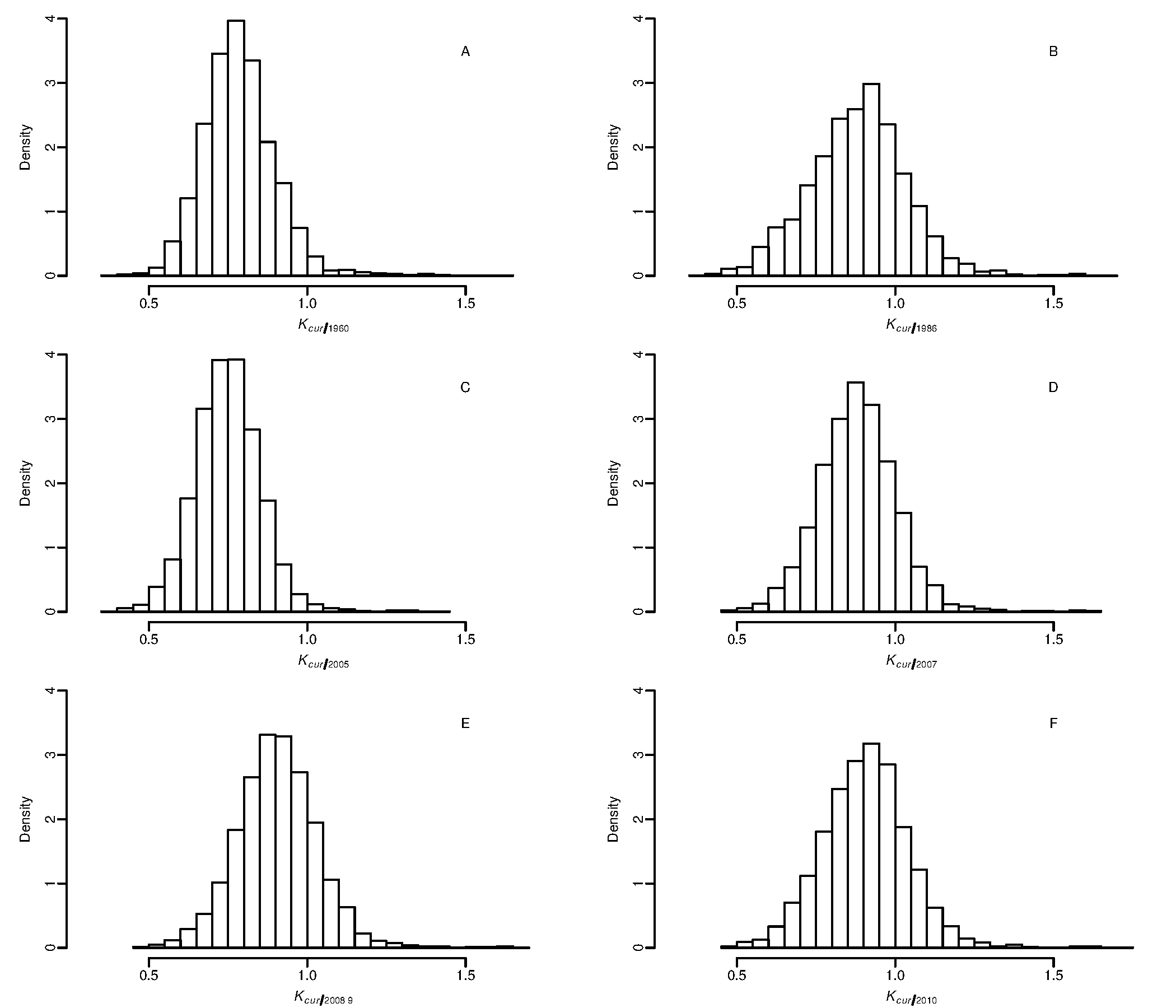

Supplement: S5 Fig — (TIF) [file pone.0171811.s005.tif]
