# Supplementary material for: Linear mixed-effects models to describe length-weight relationships for yellow croaker (Larimichthys Polyactis) along the north coast of China
Source: PLoS One. 2017 Feb 22;12(2):e0171811. doi: 10.1371/journal.pone.0171811 (PMC5321278; doi:10.1371/journal.pone.0171811)
Supplement: S1 Table — The detailed information of regions presents in Table 1; no individuals were caught during February, March and April. (DOCX) [file pone.0171811.s006.docx]

|  | Jan | May | Jun | Jul | Aug | Sep | Oct | Nov | Dec |
| --- | --- | --- | --- | --- | --- | --- | --- | --- | --- |
| YE |  |  | 8 | 6 | 8 |  | 18 |  |  |
| NS |  |  |  |  |  | 22 | 10 | 3 |  |
| JB |  | 1 |  |  | 11 | 389 | 39 | 47 | 28 |
| QD |  | 137 |  |  |  | 815 |  |  |  |
| HB |  | 433 |  | 144 | 377 | 416 | 152 |  | 93 |
| SY | 9 | 12 | 57 |  | 146 |  | 1 |  |  |
| 2008 |  |  |  |  |  | 326 | 39 | 33 | 28 |
| 2011 |  | 322 |  | 91 | 11 | 416 |  | 14 | 93 |
| 2012 |  | 129 |  |  |  | 353 |  |  |  |
| 2013 |  | 46 | 8 | 6 | 8 |  | 53 |  |  |
| 2014 |  | 57 |  | 4 | 178 | 547 | 27 | 3 |  |
| 2015 | 9 | 29 | 57 | 49 | 345 |  | 101 |  |  |
