# Supplementary material for: Linear mixed-effects models to describe length-weight relationships for yellow croaker (Larimichthys Polyactis) along the north coast of China
Source: PLoS One. 2017 Feb 22;12(2):e0171811. doi: 10.1371/journal.pone.0171811 (PMC5321278; doi:10.1371/journal.pone.0171811)
Supplement: S2 Table — See the text for the definition of Kcur/1960, Kcur/1960 and Kcur/2007. (DOCX) [file pone.0171811.s007.docx]

| *K* | References’ LWR | | Mean | Std. | 95% CI | Min. | Max. |
| --- | --- | --- | --- | --- | --- | --- | --- |
|  | ***a*** | ***b*** |  |  |  |  |  |
| *K_cur/1960_* | 0.015 | 3.110 | 0.786 | 0.115 | (0.616, 0.973) | 0.384 | 1.612 |
| *K_cur/1986_* | 0.055 | 2.525 | 0.882 | 0.154 | (0.622, 1.120) | 0.368 | 1.688 |
| *K_cur/2005_* | 0.031 | 2.835 | 0.750 | 0.107 | (0.582, 0.920) | 0.398 | 1.404 |
| *K_cur/2007_* | 0.026 | 2.840 | 0.881 | 0.125 | (0.686, 1.081) | 0.467 | 1.649 |
| *K_cur/2008 9_* | 0.027 | 2.814 | 0.906 | 0.130 | (0.700, 1.113) | 0.483 | 1.698 |
| *K_cur/2010_* | 0.034 | 2.715 | 0.906 | 0.137 | (0.683, 1.124) | 0.450 | 1.712 |
